# Survival Impact of Nephroureterectomy for De Novo Stage IV Nonmetastatic and Metastatic Upper Tract Urothelial Carcinoma

**DOI:** 10.3389/fsurg.2022.903123

**Published:** 2022-05-26

**Authors:** Wei-Yu Lin, Meng-Hung Lin, Yao-Hsu Yang, Wen-Cheng Chen, Cih-En Huang, Miao-Fen Chen, Chun-Te Wu

**Affiliations:** ^1^ Division of Urology, Department of Surgery, Chang Gung Memorial Hospital, Chiayi, Taiwan; ^2^ School of Medicine, College of Medicine, Chang Gung University, Taoyuan, Taiwan; ^3^ Department of Nursing, Chang Gung University of Science and Technology, Chiayi, Taiwan; ^4^ Department of Nursing, Shu-Zen Junior College of Medicine and Management, Kaohsiung, Taiwan; ^5^ Health Information and Epidemiology Laboratory, Chang Gung Memorial Hospital, Chiayi, Taiwan; ^6^ Department of Traditional Chinese Medicine, Chang Gung Memorial Hospital, Chiayi, Taiwan; ^7^ School of Traditional Chinese Medicine, College of Medicine, Chang Gung University, Taoyuan, Taiwan; ^8^ Department of Radiation Oncology, Chang Gung Memorial Hospital, Chiayi, Taiwan; ^9^ Division of Hematology Oncology, Department of Medicine, Chang Gung Memorial Hospital, Chiayi, Taiwan; ^10^ Division of Urology, Department of Surgery, Chang Gung Memorial Hospital, Keelung, Taiwan

**Keywords:** metastasis, nephroureterectomy, nonmetastasis, stage IV, upper tract urothelial carcinoma

## Abstract

**Background:**

Whether nephroureterectomy (NU) provides survival benefits in patients with stage IV upper tract urothelial carcinoma (UTUC) remains unclear. We compared the effect of chemotherapy (CT) alone with that of CT combined with NU (CT + NU) on the overall survival (OS) of patients with stage IV nonmetastatic UTUC (nmUTUC) and metastatic UTUC (mUTUC).

**Patients and Methods:**

This multicenter retrospective cohort study included the data of patients with UTUC undergoing CT alone or CT + NU from the Chang Gung Cancer Database (2002–2015) and followed them until August 2017. OS and hazard ratios (HRs) were assessed using the Kaplan–Meier method and Cox proportional hazards model, respectively.

**Results:**

This study included 308 patients with stage IV UTUC, comprising 139 with nmUTUC and 169 with mUTUC. Moreover, 91 (74.6%) patients with nmUTUC and 31 (25.4%) patients with mUTUC received NU. The CT + NU group had a higher 3-year OS rate (41.0.% vs 16.7%, *p *< 0.001), longer median OS duration (20.7 vs 9.0 months, *p* < 0.001), and lower risk of death (HR, 0.48; 95% confidence interval, 0.36–0.66; *p *< 0.001) than did the CT-alone group. Similarly, patients with mUTUC who underwent CT + NU had a longer median OS duration (25.0 vs 7.8 months, *p *< 0.001) and lower risk of death (HR, 0.37; 95% confidence interval, 0.23–0.59; *p* < 0.001) than did those who received CT alone.

**Conclusion:**

Compared with CT alone, NU + CT can provide survival benefits to patients with nonmetastatic and metastatic stage IV UTUC.

## Introduction

Upper tract urothelial carcinoma (UTUC) is rare and accounts for only 5%–10% of all urothelial malignancies (1). Nevertheless, approximately 10% of patients with UTUC present with locally advanced cancer or metastasis at initial diagnosis (1, 2). Furthermore, the prognosis of patients with metastatic UTUC (mUTUC) is poor, with the 3-year overall survival (OS) being <10% (1, 2). Patients with UTUC have a lower OS than do those with bladder urothelial cancer (BUC) because >60% of patients with UTUC present with invasion at diagnosis, whereas only 15%–25% of patients with BUC present with invasion at diagnosis (3, 4). Furthermore, the prognosis of patients with T4 UTUC is poor, with the 5-year OS being <10% (5, 6).

The National Comprehensive Cancer Network (NCCN) guidelines for prostate cancer, BUC, and renal cell carcinoma recommend different treatment strategies for distinct cancer stages. The recommended treatments include surgery of the primary tumor, adjuvant chemotherapy (CT), and CT with or without cytoreductive surgery. However, the recommended treatments for UTUC are relatively simple and may be outdated; such treatments are nephroureterectomy (NU) plus neoadjuvant CT (CT + NU) for selected patients with nonmetastatic UTUC (nmUTUC) and CT alone for patients with mUTUC (7). Moreover, no recommendations are specified for stage IV nmUTUC. The wide spectrum of nonmetastatic and metastatic stage IV UTUC necessitates more effective treatment strategies for this rare and lethal condition.

CT + NU has been reported to significantly prolong survival compared with either NU or CT alone in locally advanced UTUC and mUTUC (2, 8, 9). Nevertheless, evidence supporting the survival benefits of this treatment approach remains limited.

Novel immune checkpoint inhibitors have shown promising results in mUTUC, but the lack of long-term follow-up and high medical cost limit their use as the first-line treatment choice for UTUC (10). NU, the standard treatment for localized disease, is offered only palliative for patients with mUTUC (11). However, increasing bodies of evidence demonstrate survival benefits of adjuvant NU in these patients (2, 8, 12–14). To provide insights into this critical issue, we assessed the effectiveness of NU + CT compared with CT alone in providing survival benefits in patients with stage IV UTUC, including nmUTUC and mUTUC, by using the Chang Gung Research Database (CGRD).

## Methods

### Data Sources

The CGRD contains the comprehensive medical records of Chang Gung Memorial Hospital (CGMH) and includes 85 datasets. In this database, all personally identifiable information is removed to protect individual identity and privacy in accordance with strict confidentiality guidelines and personal electronic data protection regulations.

CGMH, founded in 1976, is currently the largest hospital network in Taiwan, comprising seven medical institutions. CGMH has more than 10,000 beds and admits more than 2,400,000 patients each year. Every year, the average number of the outpatient visits and surgical patients in CGMH is 8.2 million and 167,460, respectively.

### Study Population

For this multicenter retrospective cohort study, we retrieved the data of all patients with stage IV UTUC (ICD-O-3: C65–C66) from the cancer registry database within the CGRD for the period 2002–2015, and we followed them until August 2017. Patients with erroneous or missing data were excluded. We included patients who underwent CT alone (the CT-alone group) or a combination of CT and NU (CT + NU group) after being diagnosed as having stage IV UTUC, including nmUTUC (T4N0M0 or TanyN1-2M0) and mUTUC (TanyNanyM1). TNM staging was performed in accordance with AJCC, 8th edition. This study was approved by the Ethics Review Board of CGMH, Chia-Yi Branch, Taiwan (No. 201700853B0).

### Statistical Analysis

Patient characteristics were compared between groups by using the chi-square test for categorical variables and the Mann–Whitney *U* test for nonparametric data. The Kaplan–Meier method was used to calculate the OS durations, and the log-rank test was used to compare between-group differences in survival curves. In the multivariate analysis, Cox proportional hazards regression models were used to compute hazard ratios (HRs) with 95% confidence intervals (CIs) after adjustment for sex, age, clinical stage of distant metastasis (cM stage), and comorbidities (including stroke, acute myocardial infarction, chronic obstructive pulmonary disease, chronic kidney disease, and liver cirrhosis). To examine potential effect modifiers, we conducted subgroup analyses stratified by sex, age, cM stage, and comorbidities. We considered a two-sided *p* value of <0.05 as statistically significant. All analyses were conducted using SAS statistical software (Version 9.4; SAS Institute, Cary, NC, USA).

## Results

Table 1 presents the patient characteristics. We identified 308 patients with stage IV UTUC (139 with nmUTUC and 169 with mUTUC) from the CGRD. Of these patients, 186 (60.3%) underwent CT alone and 122 (39.6%) underwent CT + NU. The two treatment groups did not differ significantly in terms of sex, age, or comorbidities. However, significant between-group differences were observed in terms of cM stage and 3-year OS. Among patients with stage IV UTUC, fewer patients with mUTUC (74.6%) underwent CT + NU compared with those with nmUTUC (25.4%). The 3-year OS duration in patients with stage IV UTUC was longer in the CT + NU group than in the CT-alone group (*p* < 0.001).

**Table 1:** Baseline characteristics of patients with stage IV upper urinary tract urothelial cancer who underwent CT alone or CT + NU.

Characteristics	CT only		CT + NU		*p* value
	*n*	(%)	*n*	(%)	
Total	186		122		
Gender					0.782
Female	90	(48.4)	61	(50.0)	
Male	96	(51.6)	61	(50.0)	
Age (years)					0.009
<75	133	(71.5)	103	(84.4)	
≥75	53	(28.5)	19	(15.6)	
Median (IQR)	70	(61–76)	64	(59–71)	<0.001
Clinical stage					<0.001
T4N0M0	14	(7.5)	22	(18.0)	
TxN1M0	17	(9.1)	39	(32.0)	
TxN2M0	17	(9.1)	30	(24.6)	
TxNxM1	138	(74.2)	31	(25.4)	
cM stage					<0.001
M0	48	(25.8)	91	(74.6)	
M1	138	(74.2)	31	(25.4)	
Comorbidity					0.534
No	143	(76.9)	90	(73.8)	
Yes	43	(23.1)	32	(26.2)	
3-year survival status					<0.001
Dead	155	(83.3)	72	(59.0)	
Living	31	(16.7)	50	(41.0)	
Median (months, IQR)	9.0	(4.3–16.9)	20.7	(11.0–33.5)	<0.001

*Abbreviation: CT, chemotherapy; NU, nephroureterectomy.*

Our multivariate analysis of 3-year OS in patients with stage IV UTUC revealed that adjusted HRs (aHRs) were not significant for sex (aHR, 1.03; 95% CI, 0.79–1.34), age group (aHR, 1.33; 95% CI, 0.98–1.82), or comorbidity status (aHR, 1.01; 95% CI, 0.74–1.38). In addition, the M1 stage (aHR, 1.69; 95% CI, 1.25–2.27) was significantly associated with 3-year OS. The CT + NU group had a significantly lower risk of death (HR, 0.48; 95% CI, 0.36–0.66; *p* < 0.001) than did the CT-alone group (Table 2).

**Table 2:** Multivariate Cox regression analysis of 3-year overall survival for patients with stage IV upper urinary tract urothelial cancer.

Variables	HR_crude_	(95% CI)	*p* value	HR_adj_	(95% CI)	*p* value
Gender						
Female	Ref.			Ref.		
Male	1.15	(0.89–1.49)	0.296	1.03	(0.79–1.34)	0.841
Age (years)						
<75	Ref.			Ref.		
≥75	1.36	(1.00–1.84)	0.048	1.33	(0.98–1.82)	0.070
cM stage						
M0	Ref.			Ref.		
M1	2.19	(1.67–2.87)	<0.001	1.69	(1.25–2.27)	<0.001
Comorbidity						
No	Ref.			Ref.		
Yes	0.98	(0.72–1.34)	0.916	1.01	(0.74–1.38)	0.931
Treatment						
CT only	Ref.			Ref.		
CT + NU	0.39	(0.29–0.52)	<0.001	0.48	(0.36–0.66)	<0.001

*Abbreviation: CT, chemotherapy; NU, nephroureterectomy.*

In order to check the differences in treatment effect are not attributable to baseline imbalances between the treatment arms, we further performed the subgroup analysis to evaluate the difference and consistency. Table 3 presents the results of multivariate analysis comparing the risk of death between the CT + NU group and CT alone group stratified by gender, age, cM stage, and comorbidity status.

**Table 3:** Multivariate Cox regression analysis of 3-year overall survival in patients receiving CT + NU compared with CT alone stratified by patient characteristics.

Variables	N	HR_crude_	(95% CI)	*p* value	HR_adj_^a^	(95% CI)	*p* value
Gender							
Female	151	0.29	(0.19–0.44)	<0.001	0.35	(0.22–0.55)	<0.001
Male	157	0.51	(0.35–0.76)	<0.001	0.62	(0.41–0.96)	0.030
Age (years)							
<75	236	0.41	(0.30–0.56)	<0.001	0.49	(0.35–0.69)	<0.001
≥75	72	0.33	(0.17–0.64)	0.001	0.37	(0.17–0.80)	0.011
cM stage							
M0	139	0.61	(0.39–0.96)	0.031	0.62	(0.39–0.96)	0.034
M1	169	0.35	(0.22–0.57)	<0.001	0.37	(0.23–0.59)	<0.001
Comorbidity							
No	233	0.42	(0.30–0.58)	<0.001	0.55	(0.38–0.78)	<0.001
Yes	75	0.31	(0.17–0.55)	<0.001	0.34	(0.18–0.65)	0.001

*Abbreviation: CT, chemotherapy; NU, nephroureterectomy.*

^a^ *Hazard ratios are relative to CT alone as a reference. All estimates were adjusted for sex, age, cM stage, and comorbidity status exclusive to the specific patient population isolated for each estimate.*

In our stratified analysis of 3-year OS in the CT + NU group compared with the CT-alone group, we observed significant aHRs in patients who were women, had the M1 stage, were aged <75 years, and had comorbidities (all *p *< 0.001); we also noted significant aHRs in patients who were men, were aged ≥75 years, and had the M0 stage (*p *= 0.030, 0.011, and 0.034, respectively). Irrespective of the underlying comorbidities, the CT + NU group had a superior 3-year OS rate compared with the CT-alone group (*p *< 0.001 and 0.001, respectively; Table 3).

**Figure 1:**
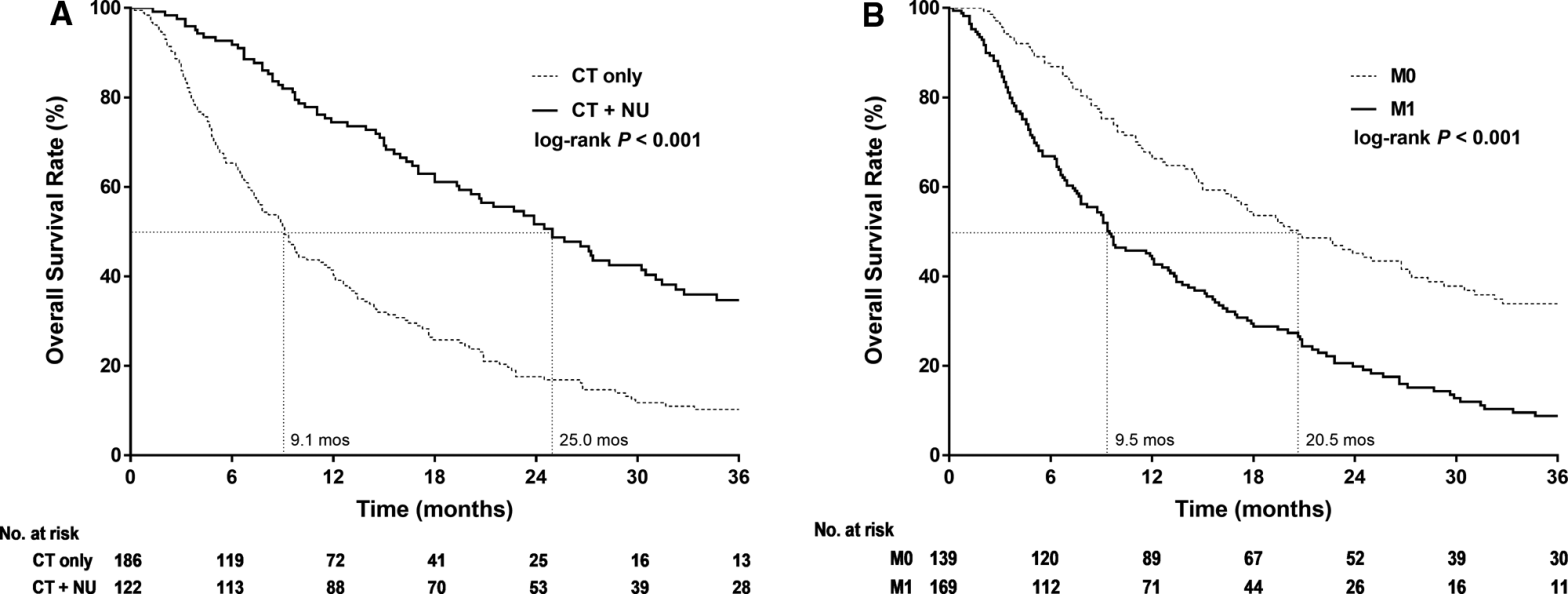
Kaplan–Meier 3-year overall survival curves for patients with stage IV upper urinary tract urothelial cancer stratified by (**A**) treatment type and (**B**) cM Stage.

The Kaplan–Meier survival curves of 3-year OS in stage IV UTUC clearly demonstrated that CT + NU provided longer survival durations than did CT alone (*p *< 0.001). The median survival durations were 9.1 and 25 months for the CT-alone and CT + NU groups, respectively (Figure 1A). Furthermore, the Kaplan–Meier survival curves of 3-year OS in the patients with stage IV UTUC clearly demonstrated that the OS duration derived for M0 was longer than that derived for M1 (*p* < 0.001, Figure 1B). The median survival durations derived for M0 and M1 were 20.6 and 9.5 months, respectively.

**Figure 2:**
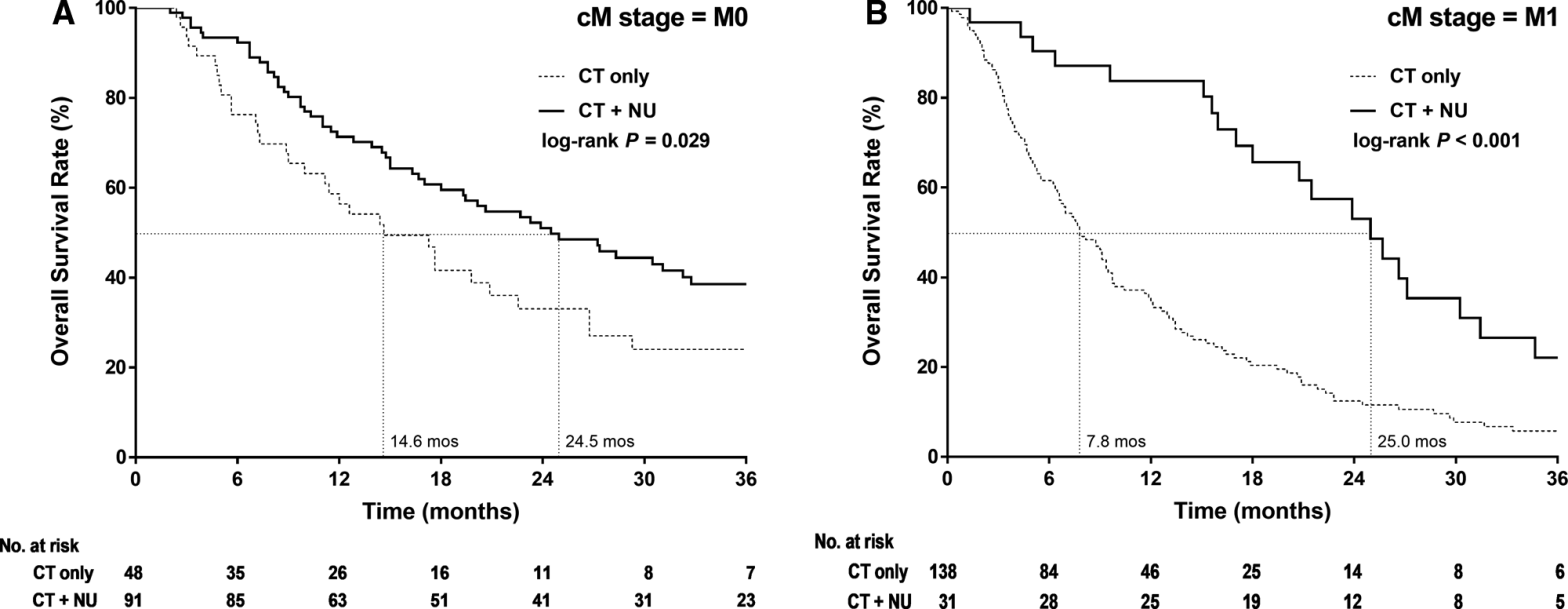
Kaplan–Meier 3-year overall survival curves for patients with stage IV upper urinary tract urothelial cancer stratified by treatment type in (**A**) nonmetastatic and (**B**) metastatic.

The Kaplan–Meier survival curves of 3-year OS in the patients with stage IV nmUTUC differed significantly between the CT-alone and CT + NU groups (*p *= 0.029, Figure 2A), with the median survival durations being 14.6 and 24.5 months, respectively. The Kaplan–Meier survival curves of 3-year OS in the patients with mUTUC demonstrated that CT + NU was significantly superior to CT alone (*p* < 0.001, Figure 2B), with the median survival duration being 25 and 7.8 months, respectively.

## Discussion

Approximately 10% of patients with UTUC present with locally advanced disease or metastasis at initial diagnosis, and the 3-year OS rates for mUTUC do not exceed 10% (1, 2). NCCN guidelines either lack treatment recommendations for stage IV nmUTUC (T4N0M0, TanyN1-2M0) or recommend CT alone for mUTUC treatment (7). Although a few studies have reported the survival benefit of CT + NU for locally advanced UTUC compared with NU alone, few have analyzed this in patients with stage IV nmUTUC (6, 9, 15–17) or mUTUC (2, 8, 13). Our study demonstrated that compared with CT alone, their combination confers OS benefits to patients with stage IV nmUTUC or mUTUC.

We observed that the median OS duration in patients with stage IV nmUTUC was longer in the CT + NU group (24.5 months) than in the CT-alone group (14.6 months). The corresponding HR was 0.62 and was statistically significant (*p *= 0.034), indicating that CT + NU was associated with a 38% reduction in the relative risk of death compared with CT alone in those patients.

Increasing numbers of studies have provided evidence that CT + NU provides survival benefits compared with NU alone for patients with locally advanced UTUC (6, 9, 15–17). However, in these studies, most of the patients had stage III disease, and only a few had stage IV disease. Although our finding is consistent with those of these studies, we compared outcomes with CT alone instead of NU alone. NU was the main treatment used for comparison in previous studies because it has been the standard therapy for locally advanced UTUC in past decades. More than one-third of patients with stage IV nmUTUC (48/139 = 34.5%) received CT alone in our study, which is lower with the study population in previous studies. The retrospective design of our study precluded the investigation of the reasons for patients receiving CT alone rather than CT + NU. Nevertheless, we speculate that this may have been due to the fact that stage IV nmUTUC differs from stage III UTUC in clinical images but is similar to mUTUC in such images, which might have influenced the clinicians’ decision to offer CT alone rather than CT + NU. Furthermore, the NCCN guidelines lack treatment recommendations for stage IV nmUTUC. Therefore, our results may serve as reference for effective treatment alternatives for this lethal and critical disease.

Although many solid cancers and urological malignancies with metastasis are treated surgically, evidence supporting the advantage of NU for stage IV mUTUC is limited (2, 8, 13, 14, 18–20). Our data indicate superior 3-year OS and a 0.37 times lower risk of death in patients with mUTUC who underwent CT + NU compared with those who underwent CT alone. Further analysis of the effect of CT + NU in patients with mUTUC, irrespective of age or comorbidities, indicated its probable benefit over CT, which is the only standard treatment for mUTUC in the NCCN guidelines (11).

Consistent with the results of previous studies, our results demonstrate CT + NU to provide a significant 3-year OS benefit compared with CT alone (2, 8, 13, 14). According to seed and soil theory, the primary tumor might activate a territory beneficial for the dissemination of metastases (21–23). Accordingly, NU might provide an additional salvage effect in patients with mUTUC. On the basis of this theory, the beneficial effect of primary tumor surgery could also be observed in metastatic BUC and metastatic renal cell carcinoma (24). These findings demonstrate that NU might provide survival benefits for mUTUC.

Our study was limited by selection bias because patients who underwent CT + NU exhibited more suitable factors, including good performance status, limited metastatic volume, and fewer comorbidities, than did their counterparts, which might have led to survival differences. Despite the lack of this information (ex: ECOG) in our study, we attempted to reduce the influence of selection bias by investigating the effects of age and comorbidities on the outcomes of the two treatments. In general, for patients with stage IV nmUTUC or mUTUC, CT + NU provided superior 3-year OS benefits compared with CT alone, regardless of age (≥75 or <75 years) or comorbidities (with or without). The Taiwanese society is aging, and older patients (age >65 years) are more frequently diagnosed as having a malignant tumor. Studies have reported that surgery can be beneficial for malignant tumors, even in patients aged >75 years (25, 26). Consistent with the results of a previous study, our results indicate the effectiveness of CT + NU therapy in older patients (≥75 years) with stage IV UTUC. Thus, old age should not be an exclusion criterion for offering NU to older patients with stage IV UTUC.

Although comorbidities are associated with poor surgical outcomes, we observed that the adjusted HR was 0.34 (*p *= 0.001) for patients with stage IV UTUC with comorbidities in the CT + NU group compared with those in the CT-alone group. The comorbidities we selected are all well-known and potent risk factors for surgery, namely stroke, acute myocardial infarction, chronic obstructive pulmonary disease, chronic kidney disease, and liver cirrhosis. Despite the underlying comorbidities in patients with stage IV UTUC, CT + NU was found to be an effective treatment for well-selected candidates compared with CT alone.

Although we could not determine whether patients in the CT + NU group underwent CT or NU first, the additional protective effect of NU compared with CT alone was evident from our results and was the focus of this study. However, future studies should analyze whether offering CT as adjuvant or neoadjuvant therapy is more beneficial in patients undergoing NU for stage IV UTUC.

## Conclusions

We used a large hospital database to examine 3-year OS in patients with stage IV nmUTUC or mUTUC who received CT alone or CT + NU. CT + NU conferred superior 3-year OS benefits for stage IV nmUTUC, and NU appeared to confer a net survival benefit for mUTUC. CT + NU could be a vital treatment consideration for suitable candidate patients with stage IV UTUC, including those with comorbidities or those aged ≥75 years.

## Data Availability

The original contributions presented in the study are included in the article/supplementary material, further inquiries can be directed to the corresponding author/s.
